# 1532. Guideline Adherence to Hepatitis B Virus (HBV) Screening and Vaccination in Patients Prescribed Human Immunodeficiency Virus (HIV) Pre-exposure Prophylaxis (PrEP)

**DOI:** 10.1093/ofid/ofad500.1367

**Published:** 2023-11-27

**Authors:** Reham S Awad, Melissa E Badowski, Sarah M Michienzi

**Affiliations:** University of Illinois Chicago, Oak Lawn, Illinois; University of Illinois Chicago, Oak Lawn, Illinois; University of Illinois Chicago College of Pharmacy, Chicago, Illinois

## Abstract

**Background:**

HIV PrEP with daily oral emtricitabine/tenofovir disoproxil fumarate (FTC/TDF) or emtricitabine/tenofovir alafenamide (FTC/TAF) has been found to be safe and effective in reducing HIV acquisition. The US Public Health Service PrEP guideline offers recommendations for the screening and vaccination for HBV in patients prescribed oral PrEP. The purpose of offering HBV screening and vaccination is twofold. One is to reduce HBV transmission, the other is to ensure appropriate treatment and monitoring for patients with HBV infection, as abrupt discontinuation of FTC/TDF and FTC/TAF could lead to an acute flare of HBV. The goal of this study was to compare guideline adherence to the rate of HBV screening and vaccination.

**Methods:**

This study was a retrospective comparative study evaluating the rate of HBV screening and vaccination in patients ≥ 18 years who received a prescription for oral PrEP at the University of Illinois Hospital and Health Sciences System (UI Health) during two time periods, 07/01/2014 - 09/30/2018 and 10/01/2018 - 10/01/2022. Patients receiving HBV treatment or with a positive HIV immunoassay blood test at baseline screening were excluded. The primary outcome compared appropriate screening and vaccination rates for HBV according to the US Public Health Service PrEP guideline. Secondary outcomes included documentation of immunity and/or vaccination to hepatitis A virus, human papilloma virus, and meningitis. The primary outcome was analyzed using a chi-square test.

**Results:**

A total of 145 patients were included in cohort 1 and served as historical control data. Of 230 patients screened for in cohort 2, 145 were included. Baseline characteristics are presented in **Table 1.** HBV screening prior to PrEP initiation occurred in 78.6% of patients in cohort 1 compared to 67.6% of patients in cohort 2 (p = 0.034). HBV vaccination was initiated by or at first follow-up in 37.9% of patients in cohort 1 compared to 21.8% of patients in cohort 2 (p = 0.035). Secondary outcomes are outlined in **Table 2.**

**Figure:**
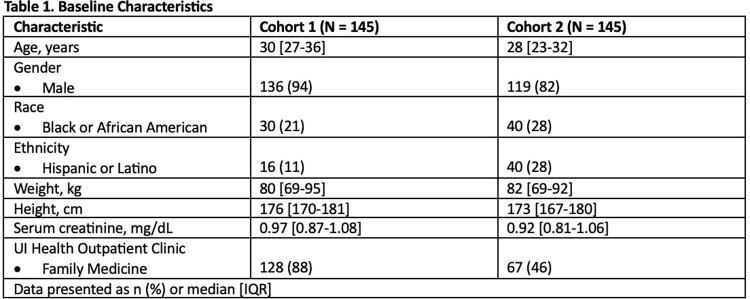

**Figure:**
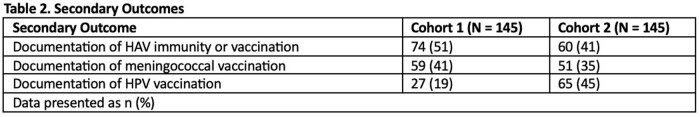

**Conclusion:**

HBV screening and rates of vaccination declined over time in patients prescribed oral PrEP. Overall, there is an increased need for education among providers prescribing oral PrEP to ensure pre-treatment HBV serologies are obtained.

**Disclosures:**

**All Authors**: No reported disclosures

